# Boosting life sciences research in Brazil: building a case for a local *Drosophila* stock center

**DOI:** 10.1590/1678-4685-GMB-2023-0202

**Published:** 2024-02-19

**Authors:** Marcos T. Oliveira, Lucas Anhezini, Helena M. Araujo, Marcus F. Oliveira, Carlos A. Couto-Lima

**Affiliations:** 1 Universidade Estadual Paulista Júlio de Mesquita Filho, Faculdade de Ciências Agrárias e Veterinárias de Jaboticabal, Departamento de Biotecnologia, Jaboticabal, SP, Brazil.; 2 Universidade Federal de Alagoas, Instituto de Ciências Biológicas e da Saúde, Departamento de Histologia e Embriologia, Maceió, AL, Brazil.; 3 Universidade Federal do Rio de Janeiro, Instituto de Ciências Biomédicas, Programa de Graduação em Biologia Celular e do Desenvolvimento, Rio de Janeiro, RJ, Brazil.; 4 Instituto Nacional de Ciência e Tecnologia em Entomologia Molecular (INCT-EM), Rio de Janeiro, RJ, Brazil.; 5 Universidade Federal do Rio de Janeiro, Instituto de Bioquímica Médica Leopoldo de Meis, Rio de Janeiro, RJ, Brazil.

**Keywords:** Genetically-defined lines, developmental biology, quantitative genetics, mitochondrial metabolism, neurobiology

## Abstract

*Drosophila melanogaster* is undoubtedly one of the most useful model organisms in biology. Initially used in solidifying the principles of heredity, and establishing the basic concepts of population genetics and of the synthetic theory of evolution, it can currently offer scientists much more: the possibility of investigating a plethora of cellular and biological mechanisms, from development and function of the immune system to animal neurogenesis, tumorigenesis and beyond. Extensive resources are available for the community of *Drosophila* researchers worldwide, including an ever-growing number of mutant, transgenic and genomically-edited lines currently carried by stock centers in North America, Europe and Asia. Here, we provide evidence for the importance of stock centers in sustaining the substantial increase in the output of *Drosophila* research worldwide in recent decades. We also discuss the challenges that Brazilian *Drosophila* scientists face to keep their research projects internationally competitive, and argue that difficulties in importing fly lines from international stock centers have significantly stalled the progression of all *Drosophila* research areas in the country. Establishing a local stock center might be the first step towards building a strong local *Drosophila* community that will likely contribute to all areas of life sciences research.

## The historical expansion of *Drosophila* research and stock centers

*Drosophila melanogaster* (Insecta: Diptera) is undoubtedly one of the most useful model organisms in biology. It is usually referred to simply as fly, fruitfly or *Drosophila* (the same terms we will use throughout this document), although the genus *Drosophila* has thousands of species and not all feed on fruits; only *D. suzukii* poses as a real threat to fruticulture as a pest (Cook and Parks, 2022). From helping to establish basic principles of genetics more than 100 years ago (Morgan, 1910), *Drosophila* then became the pupil of evolutionary biology, with a special participation of Theodosius Dobzhansky in establishing a strong research program on evolutionary population genetics in the USA and in Brazil (Glick, 2008). Dobzansky’s visits to Brazil in the 1950’s, to study tropical species drew great attention to drosophilids, and laid the foundation of strong research groups in the area of evolution and population genetics, particularly at the University of São Paulo (Magalhães and Vilela, 2014). Following the founding years in the 1950’s and 1960’s, the number of groups working with *Drosophila* has increased steadily, particularly in the areas of evolution, ecology and population genetics. Today the field of *Drosophila* research in Brazil has diversified, as revealed during the biannual Brazilian Symposium of Ecology, Genetics, and Evolution of *Drosophila* (https://wp.ufpel.edu.br/seged/historico/), but still encounters challenges in some areas.

With the advent of molecular biology, a significant increase in the use of *Drosophila* as a model organism is perceived worldwide, in great part due to the development of advanced technologies for the study of gene function in the context of the organism. *Drosophila* research on the determination of molecular mechanisms important to early animal development, on the activation of innate immunity, and on the control of circadian rhythm, are among the significant contributions this organism has provided to the science behind six Nobel Prizes in Physiology or Medicine (https://www.nobelprize.org/prizes/medicine). Many other important discoveries in biology have been pioneered by *Drosophila* research; we refer the reader to Mohr (2018) for a nearly complete list of such discoveries. Most of this science, however, was only possible because of the technological resources that have been developed for this organism throughout the years. These include, but are not limited to, an ever-growing number of mutant, transgenic and genomically-edited lines that can be used to test the role of a particular gene (virtually any gene), genomic region or genetic background in a biological phenomenon of interest. Most importantly, the community of *Drosophila* researchers worldwide can easily have access to these lines because of the establishment of facilities usually called stock centers (https://wiki.flybase.org/wiki/FlyBase:Stocks), which have the fundamental task of collecting, maintaining, creating and distributing *Drosophila* lines to any flylab in need.

Since the first *white* mutant isolated by Thomas Morgan (Morgan, 1910), thousands of other mutants have been isolated or generated for gene function studies. These include incredible genome-wide stock collections of duplication and deficiency lines essential for allelic characterization, and transgenic insertions that carry fluorescent reporters for the analysis of gene expression or to easily identify specific clones of cells *in situ*. Noteworthy are the stocks developed to help generate additional mutants, such as those carrying transposable P-element insertions (Spradling et al., 1999; Bellen et al., 2004) and the more recent MiMIC type insertions that can be swapped for a cassette of one’s choice (Venken et al., 2011; Nagarkar-Jaiswal et al., 2015), both developed as part of the *Drosophila* Gene Disruption Project (https://flypush.research.bcm.edu/pscreen/). Perhaps, one of the most important and popular tools developed for *Drosophila* research is the bipartite *UAS*/*GAL4* expression system, borrowed from the yeast *Saccharomyces cerevisiae* (Brand and Perrimon, 1993). This system relies on the transcription activator GAL4, which is temporally and/or spatially expressed in particular transgenic fly lines called drivers, and on its specificity for the *cis*-regulatory *Upstream Activating Sequence* (*UAS*) that is fused to a genetic element of interest in a distinct transgenic line. When a *GAL4* driver line is then crossed with a line bearing an *UAS* construct, the offspring may overexpress or knock down (via RNA interference, RNAi) an endogenous gene, express an exogenous gene (from a different species, including humans), a gene carrying a mutation of interest (associated with a human disease, for example) or a fusion construct to produce a fluorescently labeled protein, for instance (Caygill and Brand, 2016a , b). Throughout the years, the *UAS*/*GAL4* system has been refined, expanded and/or combined with other systems to generate more advanced analytical tools, contributing to countless work describing the roles of genes related to human biology and disease, including those important in conserved signaling pathways, neural development of the brain, tumor progression and microenvironment, among other processes (Lee and Luo, 1999; Caygill and Brand, 2016b). The North American Bloomington *Drosophila* Stock Center (BDSC), the biggest center in number of stocks carried, currently lists approximately 8,300 *GAL4* drivers, about 7,700 *UAS* non-RNAi and more than 13,000 *UAS-*RNAi stocks available (as of June, 2023, https://bdsc.indiana.edu/stocks/gal4/index.html, https://bdsc.indiana.edu/stocks/uas/index.html), illustrating the magnitude of experimental conditions possible with this system.

As for researchers working on other model systems, the *Drosophila* community worldwide has also benefited from the development and rapid expansion of the CRISPR technique (Gratz et al., 2015a ; Gratz *et al.*, 2015b) and other genome editing technologies, such that in about seven years the BDSC has accumulated and currently carries more than 4,800 related stocks for diverse purposes (as of June, 2023, https://bdsc.indiana.edu/stocks/genome_editing/index.html), including lines with the possibility of knocking out almost 3,000 genes by using a catalytically active Cas9, and of overexpressing almost 2,000 genes by targeting a nucleolytically dead Cas9 fused with the transcription activator VPR (dCas9-VPR) to a gene’s endogenous promoter. Needless to say, most of the CRISPR and other genome editing lines are dependent on the *UAS*/*GAL4* system for proper function. Remarkably, these stocks were made available almost simultaneously to their publications to the scientific community through the BDSC, highlighting once again the central roles of stock centers in making important resources widely available.

In summary, if one thinks of an experiment to examine gene, RNA and/or protein/enzyme functions in a particular cell type, tissue and/or developmental stage, it can be done efficiently and with limited financial resources using *Drosophila*. Its use as a model organism can also complement the research in the much more expensive mammalian models, where it is often impossible/particularly difficult to generate results with significance for whole-body physiology and metabolism, like it is the case for work using human cell lines in culture (Prokop, 2016; Cook and Parks, 2022). Importantly, 75-85% of genes associated with human diseases have an orthologue in *Drosophila*, and although the evolutionary lines that gave rise to insects and mammals split 500-600 million years ago, most genes retain a remarkable similarity and have conserved functions (Ugur et al., 2016; Baldridge et al., 2021; Cook and Parks, 2022). Several human diseases have been modeled in *Drosophila*, including autism spectrum disorder (Bellosta and Soldano, 2019), Alzheimer’s (Tsuda and Lim, 2018; Tue et al., 2020), Parkinson’s (Aryal and Lee, 2019), amyotrophic lateral sclerosis (Goodman and Bonini, 2020; Liguori et al., 2021), cancer (Chatterjee and Deng, 2019), diabetes (Chatterjee and Perrimon, 2021), obesity (Musselman and Kühnlein, 2018; Chatterjee and Perrimon, 2021), mitochondrial disorders (Chen et al., 2019; Rodrigues et al., 2022) and muscular dystrophies (Potikanond et al., 2018), among others. Therefore, investigating human biological processes/diseases and their associated genes in flies is not only possible, but also extremely informative. In addition to continuing providing valuable data for evolutionary genetics, *Drosophila* has now become a pre-clinical and clinical organism, in which genetic and environmental conditions can be straightforwardly tested in the context of a particular disease, and the effects of drugs and their protein/enzyme targets studied with potential medical applications. To paraphrase Cook and Parks (2022), “...these make *Drosophila* indispensable to contemporary biomedical research”. The absolute majority of the abovementioned models, if not all, are available to any researcher through the *Drosophila* stock centers in North America, Europe and/or Asia.

**Figure 1 -:**
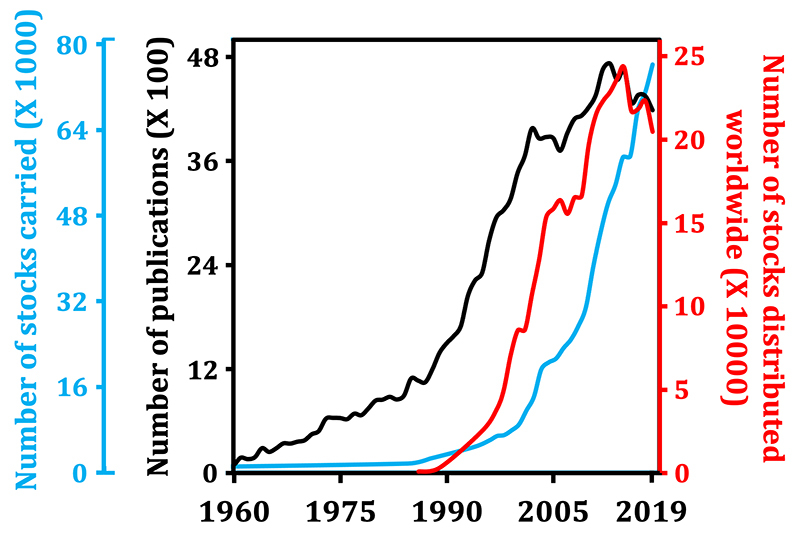
The historical increase in worldwide *Drosophila* publications is associated with the increased capacity of the BDSC to distribute its stocks. The number of publications trendline was constructed based on a search of the Scopus databank using the query “TITLE-ABS-KEY (Drosophila)”. A change in slope of this trendline can be seen after the late 1980s/early 1990s (from 1960 to 1986, linear equation y = 35.99x - 70464, R^2^ = 0.98; from 1987 to 2003, linear equation y = 191.09x - 378764, R^2^ = 0.99). See Supplementary Table S1 for raw data. The trendlines of the number of stocks carried and of stocks distributed worldwide by the BDSC were constructed based on the information available at https://bdsc.indiana.edu/about/history.html. Because of the impact of the COVID-19 pandemic on research output, data from 2020-2023 was not included.

We suspect that most Drosophilists worldwide take stock centers for granted (unwillingly perhaps), so we sought to show if maintaining and distributing the abovementioned essential fly lines around the globe have made an impact on Nobel Prize-worthy and “-almost-as-worthy” *Drosophila* research. We searched the Scopus databank (https://www.scopus.com) for worldwide publications related to *Drosophila* research throughout the years and correlated the results with the data on stocks carried and distributed by the BDSC within the same time span (https://bdsc.indiana.edu/about/history.html). Figure 1 shows how the growth in number of publications accelerated approximately 5-fold at about the same time that the BDSC started exponentially distributing its stocks. Importantly, that was just a few years prior to the first publication of the *UAS*/*GAL4* system (Brand and Perrimon, 1993). It is worth mentioning that the exponential stock distribution by the BDSC was probably boosted initially by the need of Drosophilists, who were publishing ~1000 articles per year in the 1980s (Figure 1). Curiously, both the number of publications and the number of BDSC stocks distributed have plateaued in the last 10-15 years, showing a strong association.

We also noticed that the increase in the number of BDSC stocks carried and distributed trended similarly throughout the years, except that the former lagged behind a few years and has not plateaued yet (Figure 1). The implementation of user fees in 1995 (https://bdsc.indiana.edu/about/history.html) coincided with the beginning of the expansion in the BDSC’s capacity to carry new stocks, allowing it to reach close to 80,000 stocks in 2019. Thus, an interesting positive feedback loop is apparent: the more BDSC distributed stocks, the more it contributed to *Drosophila* research output and the more revenue it generated to invest in its own capacity to carry new technologically advanced research tools Drosophilists were continuously creating. We also speculate that other stock centers have made similar impact on research output, perhaps not as much worldwide, but more locally due to their more limited size in number of stocks carried. In conclusion, stock centers are indispensable for the success of the local and worldwide *Drosophila* community in the short and long term.

## The problem of stock distribution to Brazil

For many years, we and other Brazilian *Drosophila* researchers have used the services provided by the North American, European, and Asian stock centers to obtain the fly lines necessary for our work. Despite the facilitated process of ordering from stock centers, obtaining these lines has always involved high costs and a tremendous amount of local bureaucracy, which have stalled our work significantly or prevented it from occurring even when met with the proper legal documentation. Assuming that a local Brazilian flylab is already authorized by the National Technical Committee on Biosafety (http://ctnbio.mctic.gov.br) to work with level 1 genetically modified organisms (GMOs), a simple request to the Local Biosafety Committee of the research institution to authorize the import of fly lines would in theory be sufficient, paperwork-wise. However, Brazilian customs authorities rely primarily on the documentation of another governmental agency, the Ministry of Agriculture, Livestock and Food Supply (MAPA), which is responsible for supervising the entry of live organisms into the country.

The Universal Postal Union Convention Manual (https://www.upu.int/upu/media/upu/files/upu/aboutupu/acts/manualsinthreevolumes/actinthreevolumesmanualofconventionen.pdf) is used by *Drosophila* stock centers as guidance for shipments of transgenic or non-transgenic flies to various countries. Although its Article 19 states that “flies of the family Drosophilidae for biomedical research exchanged between officially recognized institutions” are items permitted to be shipped internationally, each country can impose its own set of rules. In Brazil, MAPA’s Normative Instruction No. 36, of October 11, 2006 (http://sistemasweb.agricultura.gov.br/sislegis/action/detalhaAto.do?method=recuperarTextoAtoTematicaPortal&codigoTematica=1265040) details the operational procedures for international agricultural surveillance at ports of entry, without any specific mentioning of organisms relevant to biomedical/biological research. In many instances, *Drosophila* researchers who need to import lines for basic research often apply for the same authorizations that a large importer of agricultural goods would: a complex, arduous and bureaucratic task (details at https://sistemasweb.agricultura.gov.br/pages/SEI.html). According to the responsible office in MAPA, the official processing time of the paperwork is reasonable, but in practice we deal with professionals who are overloaded with work, untrained to assess the risks of harmless *Drosophila* lines, and often unable to approve our import requests in a timely fashion. In our experience, and that of other colleagues, the proper documents can take up to 90 days to be issued.

We recently became aware of Dispatches 579 (27459099) and 896 (27376319) from MAPA’s General Coordination of Conformity and Quality Certification (https://www.gov.br/agricultura/pt-br/assuntos/sanidade-animal-e-vegetal/saude-animal/cgccq), which state that there are no paperwork requirements for importing “insects and arthropod larvae of no veterinary importance”. The recommendation is to attach the Dispatch documents to each import of fly lines, along with the Local Biosafety Committee’s approval. Although exciting, our own consultation of the government’s list of requirements to import live animals (https://lookerstudio.google.com/u/0/reporting/2256f685-77b3-4411-9687-5ad5a4adb869/page/p_vu0f4phz3c) revealed that “insects for laboratory use”, which could also be applicable to *Drosophila*, still requires a certification signed by a veterinary doctor attesting the “good health” of the animals. In addition to the fact that no veterinary doctor in any country has likely been trained in insect health, the conflicting description of insects in official government websites and documents is confusing for scientists and for customs personnel alike, and is a source of potential denial/delay of entry during the import process. Nevertheless, Dispatches 579 and 896 might offer the fastest import route into Brazil, and must be tested from this point on for its real applicability. In the only anecdotal example that we are aware of, a fly shipment accompanied by the Local Biosafety Committee’s import approval and the abovementioned Dispatches was allowed in the country, yet with a delay. Since *Drosophila* life cycle is short (~10 days) and the shipping conditions provided by stock centers cannot properly accommodate them for much more than that time, even a short delay of a few days can compromise the delivery of live animals.

Once all import documentation is approved, Brazilian Drosophilists then face the “obstacle” of delivery. *Drosophila* stock centers usually ship their stocks in vials adequately packed in boxes, providing an appropriate environment for the flies for about 2 weeks, as mentioned above. If using standard shipping, which in the case of BDSC is via the United States Postal Services (USPS), often the stocks only arrive at labs in Brazil in 30-60 days. Even with the correct import documents, the packages remain awaiting customs approval for an excessive amount of time, often encountering warehouse temperatures well above or below what is ideal for fly survival. And because USPS relies on the Brazilian Postal Services (Correios) once the package arrives in Brazilian territory, USPS tracking stops when the package leaves the United States and Correios never registers it in its own tracking system. Thus, we often receive dead flies by mail without the option of tracking or inquiry during shipping and delivery.

Because this shipping problem likely occurs recurrently in many countries, not only Brazil, BDSC is now offering express delivery via FedEx^®^ for a flat rate. This certainly solves the problem of receiving dead flies by mail in Brazil, and helps to lower shipping costs significantly; we surely appreciate BDSC efforts on this matter. However, even with the new flat rate deal, shipping costs can still reach three times that of a purchased stock. For example, in our last purchase of BDSC stocks, we spent USD$ 15.00-25.00 per fly line for research and more than USD$ 60.00 in shipment via FedEx^®^. These numbers may not seem absurd to a researcher in the Global North, but is absolutely critical in our Brazilian perspective that combines the current funding shortage for science and the adverse exchange rate (1 BRL = ~0.2 USD). The financial limitations are amplified when considering the funds provided by main Brazilian Federal grants, which pay no more than ~USD$ 1,840.00 (BRL$ 9,166.67) per year per principal investigator (http://resultado.cnpq.br/7222512882593567; https://www.nexojornal.com.br/colunistas/2021/Qual-o-limite-de-resiliência-do-cientista-brasileiro), and the monthly stipends of PhD students with Federal funding, which were stuck at ~USD$ 440.00 (BRL$ 2,200.00) for almost a decade (Jordão, 2019; https://www.nexojornal.com.br/grafico/2022/03/23/Bolsas-da-Capes-e-CNPq-completam-9-anos-sem-reajuste); the latter just recently increased to modest ~USD$ 620.00 (BRL$ 3,100.00). Finally, upon arrival at customs, the package can still be taxed incorrectly as commercial goods, perhaps because of the conflicting description of insects in official government documents, as stated above. On one occasion, we received a single fly line as a donation from a researcher in Japan, who voluntarily paid for all FedEx^®^ shipping costs, and we were still charged ~USD$ 170.00 (BRL$ 900.00) in obscure fees by the Brazilian customs to have the entry of the flies into the country approved. The abovementioned Dispatches 579 and 896 had not yet been issued at that time, and we were admittedly not aware of how to circumvent the taxation. These fees appear to be randomly applied, as there is no clear way of knowing beforehand if a fly shipment will be tariffed upon arrival at any Brazilian port of entry.

Some science funding agencies do offer a broker service that facilitates import of scientific research material, including GMO animals. The Sao Paulo Research Foundation - FAPESP (https://fapesp.br/en), for example, is one of these agencies, but using this resource (which is provided only to FAPESP grantees) is truly only feasible when the total import costs are at least USD$ 1,000.00, as FAPESP needs to offset the costs of customs clearance. For a single Drosophilist, reaching this amount would require importing 40-65 fly lines all at once (based on the BDSC stock prices as of June, 2023 - https://bdsc.indiana.edu/order-accounts/fees/index.html), which in turn would take extensive research planning beforehand. Thus, in Brazil, we cannot order and promptly receive a few stocks to perform an experiment, like *Drosophila* researchers in developed countries usually do. Traditionally, if research funding is available to overcome the high costs, and all steps of the import process described above are completed smoothly, fly stocks will legally reach Brazilian labs in no less than 3 months. We hope the application of Dispatches 579 and 896 will expedite this for all Drosophilists, but this is yet to be shown. It is reasonable to assume that *Drosophila* researchers from other South American countries also have elevated shipping costs, difficult customs clearance processes, and/or low science funding, making fly research difficult anywhere on the continent.

**Figure 2 -:**
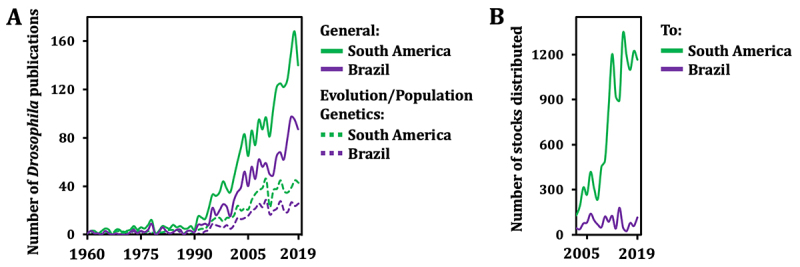
Limited BDSC stock distribution to Brazil and correlated *Drosophila* publications. The number of publications trendlines (**A**) were constructed based on a search of the Scopus databank using the queries “AFFILCOUNTRY (Brazil OR Argentina OR Colombia OR Peru OR Bolivia OR Chile OR Venezuela OR Paraguay OR Uruguay OR Ecuador) TITLE-ABS-KEY (Drosophila)”; “AFFILCOUNTRY (Brazil) TITLE-ABS-KEY (Drosophila)”; “AFFILCOUNTRY (Brazil OR Argentina OR Colombia OR Peru OR Bolivia OR Chile OR Venezuela OR Paraguay OR Uruguay OR Ecuador) TITLE-ABS-KEY (Drosophila AND evolution* OR population)”; and “AFFILCOUNTRY (Brazil) TITLE-ABS-KEY (Drosophila AND evolution* OR population). From 1990 on, linear equations for general *Drosophila* publications from South American and Brazilian only researchers were, respectively, y = 5.02x - 9981 (R^2^ = 0.95) and y = 2.97x - 5909 (R^2^ = 0.92). See Table S1 for raw data. The trendlines of the number of stocks distributed to South America and Brazil alone (**B**) were constructed based on the information made available by the BDSC to the authors. These numbers must be seen as approximations of the real number of stocks shipped to the continent due to four main reasons: 1) prior to 2011, BDSC occasionally reused account numbers, which may have produced confounding numbers; 2) the numbers do not include stocks that died en route and had to be reshipped; 3) the numbers reflect the current location of the primary BDSC account holder (the principal investigator), including from a time during which the account holder may have been based on a non-South American country; and 4) they include stocks shipped to non-South American countries, but which were ordered from a BDSC account for which the holder is based on a South American country. Because of the impact of the COVID-19 pandemic on research output, data from 2020-2023 was not included.

Directly quantifying how disruptive the high costs and bureaucracy are for Brazilian *Drosophila* research is impractical, since nuances in the relatively low budget for scientific research likely have a much bigger impact on research output. We then sought to again correlate yearly growth in number of publications by Drosophilists from Brazil and the number of BDSC stocks distributed to the country. Figure 2A shows that since 1990, publications have increased continuously in a linear fashion, with modest slope, and that Brazil is the biggest contributor of publications among South American countries. We were only able to obtain data on the BDSC stock distribution to South American countries for the last 20 years, but this appears sufficient to indicate a general tendency for increased distribution to the continent in the last ~10 years. On the other hand, no tendency for increased stock distribution to Brazil alone was observed (Figure 2B), indicating that the country’s researchers may not be taking advantage of the numerous recently developed technologies, as described in the previous section, or that other stock centers may be the primary suppliers of lines to Brazilian Drosophilists. We find that last option improbable, since the European Vienna *Drosophila* Resource Center (VDRC), perhaps the second largest stock center in the world, only lists two locations in Brazil which are their current shipping destinations of fly lines (https://www.viennabiocenter.org/vbcf/vienna-drosophila-resource-center/). When contacted recently, VDRC reported having no records of fly shipments to Brazil in the last 12 years, and only 3 shipments since its launch in 2007 (Lisa Meadows, personal communication). In summary, we cannot show that the same strong relationship observed between BDSC (or other centers) stock distribution and the worldwide output of *Drosophila* research exists in Brazil. We speculated if the “steady, but slow” increase in research output from Brazilian Drosophilists comes from studies of naturally occurring local Drosophilidae populations, which are rich in the continent (da Mata et al., 2008; Döge et al., 2008; Bizzo et al., 2010; Poppe *et al.*, 2014). Consistent with this idea and with the traditional view of Brazilian *Drosophila* research, we observed that papers on evolutionary and/or population genetics account for a third/half of the total number of *Drosophila* publications (Figure 2A). This is also consistent with our previous survey that showed that most FAPESP-funded projects using *Drosophila* are associated specifically with these areas of biology (Garcia et al., 2021).

## Limited output of many areas of *Drosophila* research from Brazil

We also explored the Web of Science databank to show the contributions since 1945 of total *Drosophila* research relative to mouse research in Brazil, the United States and Germany, as both are powerful models for the biomedical community. Our hypothesis was that the relative contribution of *Drosophila* papers in Brazil was higher than in developed countries, because of two reasons: 1) *Drosophila* research costs only a  fraction of mouse research (https://www.openaccessgovernment.org/fruit-fly-research/52396/); and 2) Brazil unfortunately shows historical, severe and pervasive financial limitations of funding science and technology, so its researchers should be more resourceful and seek more inexpensive ways for experimentation. Paradoxically, the ratio *Drosophila*/mouse research output in Brazil is remarkably lower than in the US or Germany (Table 1). This is likely influenced by a lack of knowledge on all the potential scientific and financial benefits of using flies, which serves as a barrier for biomedical researchers to adopt *Drosophila* as a model organism. The current high shipping costs of fly stocks and the difficulties at the Brazilian ports-of-entry, as discussed above, certainly elevate this barrier.

**Table 1 -:** Number of original scientific articles published in the USA, Germany and Brazil from 1945 to 2023 using mouse or *Drosophila* as model organisms.

Country	Mouse	*Drosophila*	***Drosophila* relative to Mouse (%)**
USA	500088	43124	8.6
Germany	85236	7805	9.2
Brazil	24300	1272	5.2

The number of publications was obtained from a search of the Clarivate’s Web of Science databank using the queries “mice” or “Drosophila” in the title OR abstract, and filtered for “articles” as document type, and “USA”, “Germany” or “Brazil” as country/regions.

**Figure 3 -:**
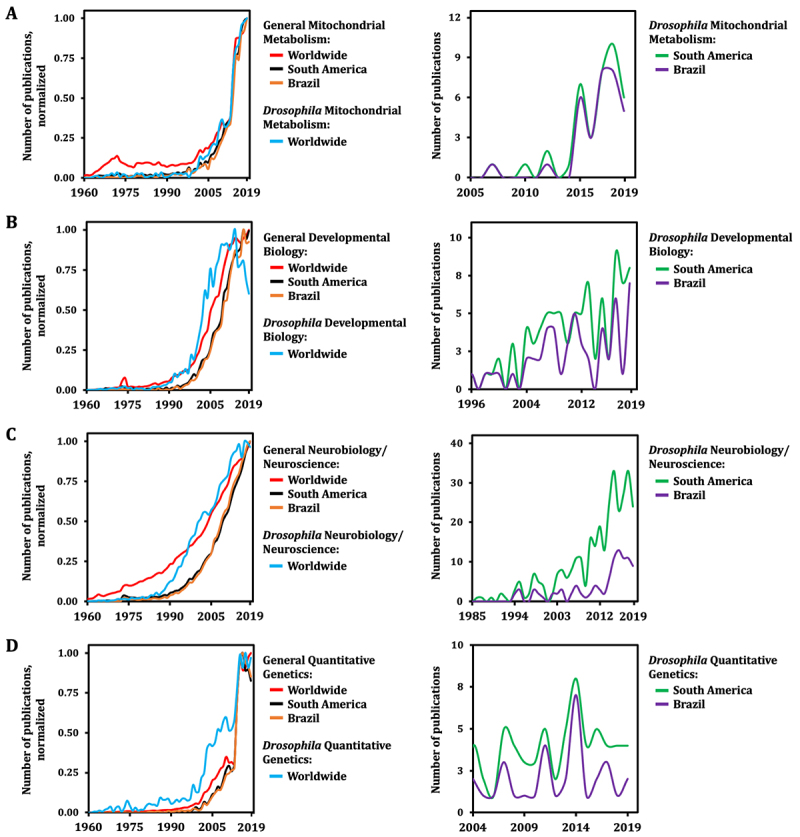
Limited output of *Drosophila* life sciences research from Brazil. **A,** The normalized trendlines for number of publications were constructed based on searches of the Scopus databank using the following queries: “TITLE-ABS-KEY (Drosophila AND mitochondr* AND metabolism)”; “TITLE-ABS-KEY (mitochondr* AND metabolism)”; “AFFILCOUNTRY (Brazil OR Argentina OR Colombia OR Peru OR Venezuela OR Chile OR Paraguay OR Uruguay OR Bolivia OR Ecuador) TITLE-ABS-KEY (mitochondr* AND metabolism)”; “AFFILCOUNTRY (Brazil) TITLE-ABS-KEY (mitochondr* AND metabolism)”; “AFFILCOUNTRY (Brazil OR Argentina OR Colombia OR Peru OR Venezuela OR Chile OR Paraguay OR Uruguay OR Bolivia OR Ecuador) TITLE-ABS-KEY (Drosophila AND mitochondr* AND metabolism)”; “AFFILCOUNTRY (Brazil) TITLE-ABS-KEY (Drosophila AND mitochondr* AND metabolism)”. The data was separately normalized by the highest yearly number of articles published, which was arbitrarily set to 1.0. See Table S1 for raw data. **B-D,** The trendlines were constructed similarly as in **A**, using queries in which the term “mitochondr* AND metabolism” was respectively replaced by “development* AND biology”, “neuro*” and “quantitative AND genetics”. Because of the impact of the COVID-19 pandemic on research output, data from 2020-2023 was not included.

We additionally investigated research output from Brazil on specific areas of biology, particularly those in which *Drosophila* is widely used worldwide. We initially focused on mitochondrial metabolism, as we are interested in its impact in human health and disease. The importance of mitochondria in *Drosophila* varies, being primarily anabolic at the larval stage to sustain the rapid growth and biomass accumulation (up to 200 fold in ~4 days at 25 ºC, Jacobs et al., 2020), and highly oxidative at the adult stage, which demands relatively high amounts of food for ATP production in its flight muscles. With the versatile research tools available and the established distribution capacities of stock centers, one would expect that the worldwide number of publications related to *Drosophila* mitochondrial metabolism research was to trend similarly to that related to general mitochondrial metabolism research, at some point after ~1993. This is in fact what we observed by exploring the Scopus databank (Figure 3A, left panel). It is also clear that the overall contribution of *Drosophila* research to the general field of mitochondrial metabolism has been nevertheless very timid (Table S1). The trends in publications on general mitochondrial metabolism in Brazil and in all South American countries combined are also very similar to that seen worldwide (Figure 3A, left panel). The contributions of Brazilian mitochondrial researchers to the field in the last 20 years have been substantial and are highlighted elsewhere (Vercesi and Oliveira, 2018). However, analyzing the output of *Drosophila* mitochondrial metabolism research from Brazil showed that no publication was recorded before 2007 and that an average of less than 3 articles on the subject were published per year since then (Figure 3A, right panel). It appears that since 2015 there has been a substantial increase in the number of publications per year in South America, mostly driven by researchers in Brazil, but the numbers are still too low to indicate a clear growth tendency and predict the overall contribution of Brazilians and other South Americans to the field of *Drosophila* mitochondrial metabolism.

We next analyzed areas in which *Drosophila* research is traditionally strong, such as developmental biology and neurobiology/neurosciences. Starting on the 1970’s and 1980’s, the field of developmental biology expanded significantly, as the astonishing genetic conservation of animal body plan determination was discovered because of the development of the first *Drosophila* molecular tools (https://www.nobelprize.org/prizes/medicine/1995/7713-the-nobel-prize-in-physiology-or-medicine-1995/). This was the first field of *Drosophila* research that was not primarily focused on inheritance, but that also used genetics and organ transplantations to investigate how genes and their products function to build animals and their parts from simpler embryos (Morata and Lawrence, 2022). *Drosophila* developmental biology is probably one of the fields that most benefited from the expansion of BDSC stock distribution capacities in early 1990’s, as it was already an intense area of research by then. We observed that research output on *Drosophila* developmental biology worldwide (Figure 3B, left panel) in fact exponentially took off concomitantly with BDSC´s distribution capacities, and followed a similar trend as that shown for general *Drosophila* publications, with a plateau in the last ~10 years (Figure 1). This trend is also quite similar to that from general developmental biology worldwide, from South America, and from Brazil alone (Figure 3B, left panel). However, once again, we observed very low yearly numbers and no clear growth tendencies on *Drosophila* developmental biology publications by Brazilians and other South Americans (Figure 3B, right panel), even though these publications started to show as early as 1996.

More recently, fly lines expressing a plethora of fluorescently tagged proteins have been developed and have been particularly useful in studying neural development, the structure and function of the adult nervous system, and behavior. *In vivo* recording and imaging specific brain cells/regions of *Drosophila* larva or adult individuals is not only possible, but also extremely informative (Zhang et al., 2010). We have learned that conserved genes regulate neuroblast polarity and cell fate in the fly and vertebrate nervous systems (Morrison and Kimble, 2006; Nakagawa et al., 2007; Boone and Doe, 2008; Bowman et al., 2008; Wu et al., 2008). In addition, amyotrophic lateral sclerosis, Parkinson’s, Alzheimer’s, and Huntington’s diseases can all be modeled in *Drosophila* adults (Tsuda and Lim, 2018; Aryal and Lee, 2019; Goodman and Bonini, 2020; Tue et al., 2020; Liguori et al., 2021), which usually show the same molecular phenotypes presented by human patients (Karvandi et al., 2023). Brazilians also appear to be missing the opportunity of exploring how *Drosophila* can contribute to the neurosciences (Figure 3C, right panel). Although the number of publications on *Drosophila* neurobiology from South Americans seems to have increased significantly in the last decade, it is still quite shy; for publications from Brazilians only, it is difficult to conclude if the small increase since 2015 is actually part of a trend (Figure 3C, right panel). These observations are not explained by a general lack of interest in neurosciences in the country, as since the late 1990’s and early 2000’s, research output by Brazilians on this area has increased dramatically, with no signs of slowing down (Figure 3C, left panel).

At last, we analyzed an area of life sciences research in which *Drosophila* has only more recently been widely adopted: quantitative genetics. The establishment of the *D. melanogaster* Genetic Reference Panel (DGRP) (Mackay et al., 2012), a collection of more than 200 inbred lines with the whole genome and transcriptome sequenced, has allowed the analyses of genome-wide association mapping to identify genes and genetic polymorphisms potentially affecting quantitative traits of interest to human biology (Jordan et al., 2012; Mackay *et al.*, 2012; Weber et al., 2012; Swarup et al., 2013; Durham et al., 2014; Arya et al., 2015; Garlapow et al., 2015; Morozova et al., 2015; Shorter et al., 2015). A user-friendly website can run the association analyses with a simple list of the quantitated phenotypes of interest (http://dgrp2.gnets.ncsu.edu/). The contribution of *Drosophila* research to quantitative genetics worldwide is such that, historically, its initial publication numbers increased more significantly than that of general quantitative genetics, although they all initiated at about the same time on the early 2000’s (Figure 3D, left panel). Research output on general quantitative genetics from Brazil trended similarly to the worldwide data (Figure 3D, left panel), but we observed once again no clear trends for publications on *Drosophila* quantitative genetics from the country (Figure 3D, right panel). In conclusion, to keep pace with the international *Drosophila* community, contributing significantly to the different fields of the life sciences, efforts to facilitate the use of *Drosophila* as a model organism in the country are unquestionably required.

## Conclusions: A way to boost *Drosophila* and general life sciences research in Brazil

Here, we have shown the historical intimate relationship between stock distribution by the BDSC and the increase in worldwide *Drosophila* research output, as measured by the yearly number of publications registered in the Scopus databank since 1960. We have also seen in the Web of Science databank that the contribution of fly research relative to mouse research in Brazil is about half of that in the USA and Germany, which is counterintuitive considering the availability of science funds in these countries and the relative costs of fly and mouse research. Additionally, we have shown that BDSC stock distribution to Brazil is limited and that it appears to have no clear relation with the number of *Drosophila* publications by Brazilian researchers. When we evaluate only publications on *Drosophila* mitochondrial metabolism, one of our areas of interest, the numbers are simply too low to infer any clear pattern or correlation, even though mitochondrial metabolism and redox biology in the country are firmly established fields of research. These findings are also true for areas of research in which *Drosophila* has been traditionally used as an important model organism, such as developmental biology and neurobiology, and for more recently developed areas, such as quantitative genetics. We also presented anecdotal evidence that one of the most important impediments for the expansion of *Drosophila* research in Brazil is the bureaucracy and high shipping costs associated with the importation of GMO fly lines into the country. Small individual *Drosophila* research groups usually have low leverage, such that dealing with fly line imports often becomes too much of a burden, even when research funds are available. This may ultimately discourage a lab from starting to use *Drosophila* as their main model organism for biomedical/biological research, despite the myriad genetic tools this organism offers.

The difficulties of importing reagents/materials for scientific research in Brazil is not exclusive to Drosophilists. This is a long-lasting problem that has affected scientists in all areas, caused by an inherently inefficient customs service (https://revistapesquisa.fapesp.br/en/supply-side-research-constraints/). We find it improbable that this system will be reasonably improved in the near future, or that our currently small community of *Drosophila* researchers will gain enough leverage to establish a channeled fly import process through a customs broker service. We first need to make the community grow, but this cannot be achieved without a fast way to receive the fly lines of interest. Therefore, to boost general *Drosophila* research in Brazil, and possibly in all South America, we propose the establishment of a local stock center that will be able to maintain and distribute important fly stocks all over the country affordably and reliably. Given the trajectory of BDSC stock distribution and its correlation with the increase in worldwide *Drosophila* publications (Figure 1), we envision that a local South American stock center will likely be the most important step towards the construction of a foundation upon which the *Drosophila* community will expand significantly in a matter of years. We will also trust our currently small community to advocate for *Drosophila* research in their own local workplaces and in national and international conferences.

The establishment of a local stock center will come with no shortage of challenges. The development of new genetic tools, such as a collection of *UAS* non-RNAi, *UAS*-RNAi or of CRISPR lines that would be exclusive to the new stock center, would demand a tremendous effort and a significantly high investment. Instead, we envision the possibility of creating a “branch” of the North American, European and/or Asian stock centers, in which some of their already well-established stocks would be made available once to our future Brazilian/South American stock center, to be kept locally, indefinitely and always ready to be shipped promptly, for an affordable price. All the importation troubles described above would then be concentrated and only occur once every one or two years, when the local stock center would expand by receiving new stocks from their original sources. This expansion would be done as democratically as possible by consulting the *Drosophila* community about their fly lines of interest. Some of the well-established stock centers may find this proposition to be not worth pursuing, since most of the revenue generated by stock distribution by the putative future Brazilian/South American stock center would remain locally; only a fraction of the revenue would then be paid to the center in which the stocks originated, as some sort of “royalty” fee. We believe that in the long run distributing stocks to Brazilian/South American labs mediated by a local “branch” will pay off to the original stock centers; the alternative would be to continue receiving a minimal number of orders (or none at all) and very little revenue from Brazilians due to either the small size of our fly community or the impracticalities of our importation system.

The establishment of this future stock center in Brazil may impose other types of challenges. Brazil is arguably the number one country in enforcing its rights under the Nagoya Protocol on Access to Genetic Resources and the Fair and Equitable Sharing of Benefits Arising from their Utilization to the Convention on Biological Diversity (https://treaties.un.org/pages/ViewDetails.aspx?src=IND&mtdsg_no=XXVII-8-b&chapter=27&clang=_en#EndDec), because it is legitimately concerned about pharmaceutical industries and other countries exploiting its natural resources while seeing no economic benefits from their use. The country adopts a broad interpretation of “natural resource”, but at this point it is not clear if that comprises research organisms such as *Drosophila*. In theory, it is possible that the authorities may view any fly line originated elsewhere that has been propagated in a lab in Brazil as subject to the Nagoya Protocol, even if it is unchanged genetically. This may be an impediment/discouragement for stocks to leave the center in Brazil towards other South American countries, or at least, the issue of GMO flies originated elsewhere being under the Nagoya Protocol in Brazil would have to be discussed and clarified with government authorities prior to the shipping of the first stocks out of the country.

The new stock center would have to be attractive to researchers, showing the advantages of using *Drosophila* as their main or additional model organism. Once again, we will also have to count on our community to advocate for fly research and help show such advantages. But, in our opinion, the abundance of genetic tools provided by *Drosophila* will not necessarily tip the balance towards a switch in model organisms by a research group if it does not come associated with lower costs, and a change in culture, especially of the local biomedical community. We are currently quantifying how much it would cost to perform several different types of experiments commonly executed in a molecular biology lab in Brazil using *Drosophila* and human cells in culture, with the intention to also show financial advantages for the former and to help create attractiveness. Nevertheless, once established, the putative new Brazilian/South American stock center should rely primarily (or even solely) on funds from universities, research institutions and funding agencies during its first years, balancing the costs of stock maintenance and distribution, and providing fly lines at a minimal price (or even free of charge) to local Drosophilists, making the use of *Drosophila* very appealing. Government funding of the putative stock center will then be key, as it is even for the well-established BDSC (https://bdsc.indiana.edu/about/funding.html) and VDRC (https://shop.vbc.ac.at/vdrc_store/funding-sources). One frequently hears that flying is most often the fastest and sometimes the cheapest way to reach a destination. For life science researchers from Brazil and other South American countries, we believe it is time to “fly”!

## Supplementary material

The following online material is available for this article:

Table S1 - Yearly number of publications (since 1960) of the indicated life science areas contributed by researchers worldwide, from South America and from Brazil.
