# Supplementary material for: Boosting life sciences research in Brazil: building a case for a local Drosophila stock center
Source: Genet Mol Biol. 2024 Feb 19;47(1):e20230202. doi: 10.1590/1678-4685-GMB-2023-0202 (PMC10917079; doi:10.1590/1678-4685-GMB-2023-0202)
Supplement: Table S1 - [file 1415-4757-GMB-47-1-e20230202-s1.pdf]

Genetics and Molecular Biology
https://doi.org/10.1590/1678-4685-GMB-2023-0202

Supplementary Material to “Boosting life sciences research in Brazil: building a case for a local Drosophila stock center”

Table S1 - Yearly number of publications (since 1960) of the indicated life science areas contributed by researchers worldwide, from South America and from Brazil.

	Drosophila			***Drosophila* Evolutionary/Pop. Genetics**		**General Mitochondrial Metabolism**			***Drosophila* Mitochondrial Metabolism**			**General Developmental Biology**		
**Year**	**Worldwide**	**South America**	**Brazil**	**South America**	**Brazil**	**Worldwide**	**South America**	**Brazil**	**Worldwide**	**South America**	**Brazil**	**Worldwide**	**South America**	**Brazil**
2019	4186	140	87	43	26	12580	581	405	172	6	5	11180	512	315
2018	4347	168	95	45	24	11600	571	373	168	10	8	10798	486	313
2017	4363	150	97	39	27	11303	539	366	164	8	8	10608	516	341
2016	4273	128	79	35	19	11022	457	299	144	3	3	10291	459	287
2015	4643	122	62	36	20	10950	441	310	139	7	6	10480	447	282
2014	4537	125	68	45	28	8553	342	208	117	1	0	10640	436	297
2013	4724	122	65	38	22	4800	209	137	66	0	0	10215	413	269
2012	4657	103	49	37	20	4397	202	130	57	2	1	10136	377	238
2011	4360	81	50	23	17	3999	187	112	55	0	0	9441	330	192
2010	4219	97	59	46	29	4357	145	95	63	1	0	8649	312	188
2009	4123	87	56	39	23	3752	134	79	55	0	0	7645	231	140
2008	4084	95	62	37	26	3456	110	60	36	0	0	6622	205	129
2007	3923	74	46	34	22	2876	89	47	37	1	1	6479	192	124
2006	3719	86	56	29	20	2651	82	49	33	0	0	5998	180	107
2005	3865	65	40	21	16	2316	56	22	25	0	0	5464	133	84
2004	3876	83	52	22	14	2325	60	34	23	0	0	4227	109	68
2003	3861	72	39	20	13	1991	44	32	20	0	0	3901	100	65
2002	3974	60	35	24	14	2171	55	25	23	0	0	3503	95	49
2001	3656	47	28	16	8	1754	34	23	10	0	0	2761	69	41
2000	3464	35	15	14	5	1365	32	14	8	0	0	2369	45	27
1999	3161	38	23	14	8	1168	19	12	6	0	0	1922	51	21
1998	3031	44	25	11	6	1146	38	23	1	0	0	1713	37	21

	Drosophila			***Drosophila* Evolutionary/Pop. Genetics**		**General Mitochondrial Metabolism**			***Drosophila* Mitochondrial Metabolism**			**General Developmental Biology**		
**Year**	**Worldwide**	**South America**	**Brazil**	**South America**	**Brazil**	**Worldwide**	**South America**	**Brazil**	**Worldwide**	**South America**	**Brazil**	**Worldwide**	**South America**	**Brazil**
1997	2948	35	20	14	7	1074	22	13	5	0	0	1432	24	13
1996	2689	32	16	14	8	1082	20	10	4	0	0	1336	19	11
1995	2309	33	22	12	9	1067	16	6	5	0	0	1319	15	7
1994	2223	22	8	9	4	980	20	9	4	0	0	1097	10	3
1993	2046	13	9	5	3	942	16	8	5	0	0	1063	14	8
1992	1695	14	8	5	1	1005	16	10	4	0	0	996	7	6
1991	1581	15	8	4	1	939	12	6	2	0	0	864	7	2
1990	1498	4	2	0	0	848	12	5	1	0	0	628	2	1
1989	1388	7	3	1	0	954	14	5	1	0	0	603	8	2
1988	1198	5	3	3	1	983	17	8	6	0	0	557	2	0
1987	1049	5	0	1	0	1209	10	3	2	0	0	470	4	1
1986	1067	7	4	4	2	1056	10	1	5	0	0	396	6	4
1985	1092	6	4	4	4	1027	7	3	2	0	0	480	3	0
1984	895	8	2	4	1	1147	12	5	2	0	0	429	2	1
1983	850	5	1	4	1	1191	8	3	1	0	0	335	4	2
1982	880	6	3	3	1	1173	11	2	1	0	0	281	4	1
1981	848	7	6	2	1	1112	11	4	5	0	0	261	1	0
1980	837	3	2	2	2	1151	9	5	4	0	0	227	3	1
1979	748	1	0	1	0	1123	11	4	5	0	0	249	1	1
1978	669	12	9	2	2	816	6	2	2	0	0	198	1	0
1977	686	8	4	3	0	882	7	4	1	0	0	235	5	1
1976	623	5	2	1	1	918	5	2	0	0	0	253	2	0
1975	636	6	3	3	3	1051	8	1	3	0	0	240	4	1
1974	638	4	1	3	1	1154	4	0	4	0	0	860	6	2
1973	628	7	4	5	3	1299	15	2	1	0	0	670	12	2
1972	486	4	2	2	1	1707	14	0	6	0	0	227	5	1
1971	448	3	0	0	0	1525	9	0	3	0	0	181	1	1

	Drosophila			*Drosophila* Evolutionary/Pop. Genetics		General Mitochondrial Metabolism			*Drosophila* Mitochondrial Metabolism			General Developmental Biology		
**Year**	**Worldwide**	**South America**	**Brazil**	**South America**	**Brazil**	**Worldwide**	**South America**	**Brazil**	**Worldwide**	**South America**	**Brazil**	**Worldwide**	**South America**	**Brazil**
1970	379	1	0	0	0	1470	9	1	5	0	0	170	0	0
1969	363	4	0	3	0	1231	7	2	3	0	0	123	3	2
1968	341	4	1	0	0	1085	8	0	3	0	0	143	1	0
1967	343	0	0	0	0	944	7	0	1	0	0	115	6	1
1966	286	4	1	2	1	740	8	2	0	0	0	84	1	0
1965	245	5	3	2	1	551	8	1	2	0	0	63	0	0
1964	288	3	0	1	0	450	5	4	2	0	0	71	1	0
1963	184	1	1	1	1	255	1	1	1	0	0	32	0	0
1962	162	3	1	1	0	181	0	0	0	0	0	21	0	0
1961	178	3	3	3	3	206	1	1	0	0	0	11	0	0
1960	95	1	1	1	1	171	0	0	0	0	0	11	0	0

Table S1 continued.

	Drosophila Developmental Biology			General Neurobiology/ Neuroscience			*Drosophila* Neurobiology/ Neuroscience			General Quantitative Genetics			*Drosophila* Quantitative Genetics		
**Year**	**Worldwide**	**South America**	**Brazil**	**Worldwide**	**South America**	**Brazil**	**Worldwide**	**South America**	**Brazil**	**Worldwide**	**South America**	**Brazil**	**Worldwide**	**South America**	**Brazil**
2019	165	8	7	148361	5947	3841	1180	24	9	13898	424	305	156	4	2
2018	188	7	1	141375	5756	3748	1214	33	11	13495	461	329	146	4	1
2017	221	9	6	136185	5352	3518	1225	27	11	12611	457	336	161	4	3
2016	215	2	2	132444	4924	3339	1101	23	13	12417	514	358	146	5	2
2015	211	6	4	130611	4620	3084	1202	33	11	13527	488	343	159	4	1
2014	274	2	0	127415	4374	2955	1154	25	6	9994	369	257	126	8	7
2013	256	7	2	124939	4116	2814	1134	13	2	4228	154	100	94	5	2
2012	240	5	3	118748	3742	2581	1088	19	3	4395	133	95	84	2	1
2011	251	5	5	109345	3359	2266	971	14	4	4352	151	88	83	5	4
2010	248	3	3	104498	3174	2178	939	16	2	4853	138	84	96	3	1
2009	249	5	1	100414	2950	2025	916	4	1	4048	106	66	92	3	1

	Drosophila Developmental Biology			General Neurobiology/ Neuroscience			*Drosophila* Neurobiology/ Neuroscience			General Quantitative Genetics			*Drosophila* Quantitative Genetics		
**Year**	**Worldwide**	**South America**	**Brazil**	**Worldwide**	**South America**	**Brazil**	**Worldwide**	**South America**	**Brazil**	**Worldwide**	**South America**	**Brazil**	**Worldwide**	**South America**	**Brazil**
2008	224	5	4	94203	2562	1772	884	11	2	3811	79	51	82	4	1
2007	209	5	4	90349	2288	1564	790	11	4	3344	84	51	84	5	3
2006	177	4	2	86436	2173	1476	730	8	2	2947	64	44	71	1	1
2005	208	3	2	82061	1811	1167	701	6	0	2786	59	36	67	2	1
2004	158	4	2	75496	1704	1065	662	8	3	2178	40	26	69	4	2
2003	170	0	0	69465	1574	979	682	7	2	1853	28	17	64	0	0
2002	123	3	1	64716	1391	849	678	2	2	1692	35	21	40	0	0
2001	96	0	0	60853	1174	714	629	0	0	1155	16	8	35	0	0
2000	72	2	1	58792	1110	637	599	4	1	760	11	5	21	0	0
1999	52	1	1	55604	986	553	514	5	2	786	18	8	21	0	0
1998	60	1	1	52467	830	459	492	7	3	648	10	9	28	0	0
1997	29	0	0	51282	838	456	454	2	0	545	5	4	14	0	0
1996	36	1	1	47532	668	373	362	1	0	431	6	5	12	0	0
1995	29	0	0	45897	559	304	334	5	3	427	5	3	14	0	0
1994	30	0	0	44055	488	258	271	3	2	382	6	5	14	0	0
1993	22	0	0	41867	409	202	260	0	0	324	3	3	11	0	0
1992	28	0	0	39643	397	213	207	1	0	313	6	4	13	0	0
1991	14	0	0	35312	345	169	162	2	0	256	2	2	10	0	0
1990	13	0	0	34682	318	151	152	0	0	209	2	2	9	0	0
1989	9	0	0	31235	227	90	128	1	0	212	5	4	14	0	0
1988	8	0	0	28899	242	111	90	0	0	206	1	1	13	0	0
1987	6	0	0	26485	200	80	70	1	0	198	1	0	13	0	0
1986	6	0	0	26691	163	64	49	1	0	169	2	2	15	0	0
1985	5	0	0	25610	172	74	66	0	0	133	3	3	7	0	0
1984	5	0	0	23675	174	80	45	0	0	157	1	1	8	0	0
1983	1	0	0	22117	165	59	47	1	1	151	2	2	8	0	0
1982	0	0	0	21680	149	61	28	0	0	112	2	2	9	0	0
1981	1	0	0	20403	138	54	23	0	0	99	0	0	7	0	0

	Drosophila Developmental Biology			General Neurobiology/ Neuroscience			*Drosophila* Neurobiology/ Neuroscience			General Quantitative Genetics			*Drosophila* Quantitative Genetics		
**Year**	**Worldwide**	**South America**	**Brazil**	**Worldwide**	**South America**	**Brazil**	**Worldwide**	**South America**	**Brazil**	**Worldwide**	**South America**	**Brazil**	**Worldwide**	**South America**	**Brazil**
1980	3	0	0	18892	152	56	34	0	0	93	0	0	3	0	0
1979	2	0	0	18566	134	44	24	0	0	98	0	0	3	0	0
1978	3	0	0	17056	124	43	23	0	0	85	0	0	2	0	0
1977	0	0	0	15793	143	58	21	0	0	77	0	0	5	0	0
1976	4	0	0	14885	147	63	27	0	0	71	0	0	2	0	0
1975	3	0	0	14862	155	70	14	0	0	98	0	0	7	0	0
1974	6	0	0	15453	202	88	18	0	0	107	1	0	12	0	0
1973	5	0	0	14171	219	86	19	0	0	77	1	1	5	0	0
1972	4	0	0	10027	57	10	16	0	0	69	1	1	1	0	0
1971	2	0	0	9210	53	16	18	0	0	57	0	0	6	0	0
1970	3	0	0	8318	39	5	9	0	0	42	0	0	5	0	0
1969	3	0	0	7850	30	6	7	0	0	39	1	0	1	1	0
1968	2	0	0	7106	38	2	5	0	0	54	1	1	6	0	0
1967	1	0	0	6233	37	5	2	0	0	37	0	0	4	0	0
1966	1	0	0	5160	28	3	7	0	0	18	0	0	3	0	0
1965	2	0	0	5565	27	6	4	0	0	24	0	0	1	0	0
1964	1	0	0	6663	17	4	7	0	0	26	0	0	2	0	0
1963	0	0	0	4846	13	3	1	0	0	11	0	0	1	0	0
1962	0	0	0	3147	15	3	1	0	0	7	0	0	1	0	0
1961	0	0	0	2700	7	3	0	0	0	8	0	0	0	0	0
1960	0	0	0	2496	5	2	1	0	0	3	0	0	0	0	0
